# High-Dose Chemotherapy Followed by Autologous Stem Cell Transplantation as a First-Line Therapy for High-Risk Primary Breast Cancer: A Meta-Analysis

**DOI:** 10.1371/journal.pone.0033388

**Published:** 2012-03-12

**Authors:** Jing Wang, Qiguo Zhang, Rongfu Zhou, Bing Chen, Jian Ouyang

**Affiliations:** 1 Department of Hematology, the Affiliated DrumTower Hospital of Nanjing University Medical School, Nanjing, People's Republic of China; 2 Center of Bone Marrow Transplantation, the Affiliated DrumTower Hospital of Nanjing University Medical School, Nanjing, People's Republic of China; Copenhagen University Hospital Gentofte, Denmark

## Abstract

**Background and Objectives:**

Several trials have generated conflicting results about the results of high-dose chemotherapy followed by autologous stem cell transplantation (HDCT) for primary breast cancer. This meta-analysis summarizes the available evidence from all suitable studies.

**Design and Methods:**

Prospective, randomized trials with HDCT as a first-line therapy for primary breast cancer were included in this meta-analysis. The primary outcome of interest for our analysis was survival (disease-free survival and overall survival); secondary endpoints included treatment-related mortality (TRM) and second (non-breast) cancers. We used a median age of 47, a PR positive rate of 50% and a premenopausal rate of 70% as cutoff values to complete the subgroup analyses, which were pre-planned according to the prepared protocol.

**Results:**

Fourteen trials with 5747 patients were eligible for the meta-analysis. Compared with non-HDCT, non-significant second (non-breast) cancers (RR = 1.28; 95% CI = 0.82–1.98) and higher TRM (RR = 3.42; 95% CI = 1.32–8.86) were associated with HDCT for primary breast cancer. A significant DFS benefit of HDCT was documented (HR = 0.89; 95% CI = 0.79–0.99). No difference in OS (overall survival) was found when the studies were pooled (HR = 0.91; 95% CI = 0.82–1.00, *p* = 0.062). In subgroup analysis, age and hormone receptor status had a significant interaction with prolonged DFS and OS.

**Conclusions:**

HDCT has a benefit on DFS and OS compared to SDC in some special patients with high-risk primary breast cancer.

## Introduction

In 2010, breast cancer was ranked first in cancer incidence among women in US, with an estimated 207,090 cases. For cancer-related mortality, breast cancer was ranked second among women, with an estimated 39,840 deaths [1]. Patients with stage III breast cancer or patients with stage II breast cancer and multiple positive axillary lymph nodes have an approximately 80% relapse rate at 5 years if treated only with locoregional therapy [2]–[4]. One of the strategies to improve the outcome for high- risk patients was to increase the dose of chemotherapy to enhance its cytotoxicity. The technique of high-dose chemotherapy followed by autologous stem cell transplantation (HDCT) has been considered an exciting development because, by addressing the problem of bone-marrow toxicity, it permits the administration of doses many times higher than could otherwise be considered and thus results in the death of more tumor cells. Therefore, most research on breast cancer management focuses on improving breast cancer outcomes in this area.

A prospective randomized clinical trial is the accepted standard for comparing different treatments, such as different treatments for primary breast cancer. Many randomized trials performed by several institutions across the world have addressed conflicting results regarding the benefit of HDCT for primary breast cancer. A large meta-analysis of 13 randomized trials including 5,064 women, showed a significant benefit in event-free survival for the HDCT group, and overall survival rates were not significantly different at any stage of follow-up [5]. However, individual patient data from 15 known randomized trials including 6,210 patients showed a modest improvement in OS (HR 0.89; 95% CI 0.81–0.98; P = 0.016) for the HDCT group compared with standard dose chemotherapy (SDC) [6]. It became unclear whether HDCT results in a survival benefit compared with SDC. To arrive at comprehensive estimates of the survival benefit from the totality of the data available, we performed a meta-analysis of all relevant randomized trials that compared HDCT with SDC in patients with primary breast cancer in order to search for the proper subgroup of patients who will benefit from this kind of treatment. It was performed in accordance with the Preferred Reporting Items for Systematic reviews and Meta-Analyses (PRISMA) guidelines [7]. The protocol for this trial and supporting PRISMA checklist are available as supporting information; see Checklist S1 and Protocol S1.

## Methods

### Search strategy

The Cochrane Controlled Trials Register, MEDLINE and EMBASE were searched until March 2010. The publication type term was *Randomized Controlled Trial*. No other restrictions were applied. Additionally, reference lists of all identified trials and of comprehensive reviews in the field were screened. The volumes of abstracts of the annual meetings of the American Society of Hematology (ASH), the European Haematology Association (EHA), and the American Society of Oncology (ASCO) were screened from 1995 to 2010.

### Inclusion and exclusion criteria

For inclusion, the trials had to be prospective and randomized with standard conventional chemotherapy in one arm compared with high-dose chemotherapy followed by autologous stem cell transplantation in the other arm as first-line therapy of patients with primary breast cancer. Trials not fulfilling the inclusion criteria were excluded.

### Extraction process

A structured form was used to extract relevant data from the trials. Extraction was performed independently by two reviewers. Disagreements were resolved by consensus. Reviewers were not blinded to availability, as abstracts were obtained personally.

### Outcome and definition

The primary outcome of interest for our analysis was survival (disease-free survival and overall survival); secondary endpoints included treatment-related mortality (TRM) and second (non-breast) cancers. The above information was extracted from each study. We did not define any minimum number of patients to include a study in our meta-analysis.

### Statistical analysis

To estimate the treatment effects, outcomes were calculated as either relative risks (RRs) or hazard ratios (HRs), with their respective 95% confidence intervals (CIs) (a benefit of HDCT would be represented by an HR or RR<1). Survival outcome data were synthesized using the time-to-event hazard ratio as the effect measure. When HRs were not given in a paper, data were extracted from the respective Kaplan-Meier curves to calculate HRs [8]. Heterogeneity was checked by a Q-test. A *p* value of more than 0.10 for the Q-test indicates a lack of heterogeneity across trials. Considering the inherited heterogeneity between these studies, we assumed the presence of statistical heterogeneity and decided to use a random effects model before pooling the data. Evidence of publication bias was determined using the methods of Egger et al. and Begg et al. Moreover, contour-enhanced funnel plotting was performed to aid the interpretation of the funnel plot [9]. Tests of interaction across the subgroups were performed to assess whether the benefit of HDCT varied significantly among patients of different conditions. Review Manager (Version 5.0 for Windows) and STATA 10.0 were used for the statistical analysis.

### Subgroup analysis

Subgroup analysis was conducted in an effort to determine whether modification of the inclusion criteria of this meta-analysis affected the final results. The median patient age was 46 years, hormone receptor (PR) status was positive in 46.8% of patients, and 68.9% of patients were premenopausal in the overall population [6]. We performed the subgroup analysis, which were pre-planned according to the prepared protocol for this meta-analysis, by limiting the meta-analysis to studies using the following criteria (Table 1): (a) Median age <47 in each group; (b) PR positive (positive if either estrogen or progesterone receptor was positive) rate >50% in each group; and (c) Premenopausal rate >70% in each group.

**Table 1:** Subgroups according to patient characteristics.

Patient characteristics	IBCSG	SBG	ICCG	MDACC	ACCOG	SWOG	WSG	CALGB	PEGASE01	NWAST	Dutch pilot	ECOG	JCOG	GABG
Median age <47 in each group	+	−	−	+	+	0	−	+	+	+	−	+	−	0
PR positive rate >50% in each group*	−	−	+	−	−	+	+	+	+	+	−	+	+	+
Premenopausal rate >70% in each group	−	0	−	0	0	+	−	+	−	+	+	+	+	−

*PR positive: if either estrogen or progesterone receptor was positive.

## Results

**Figure 1:**
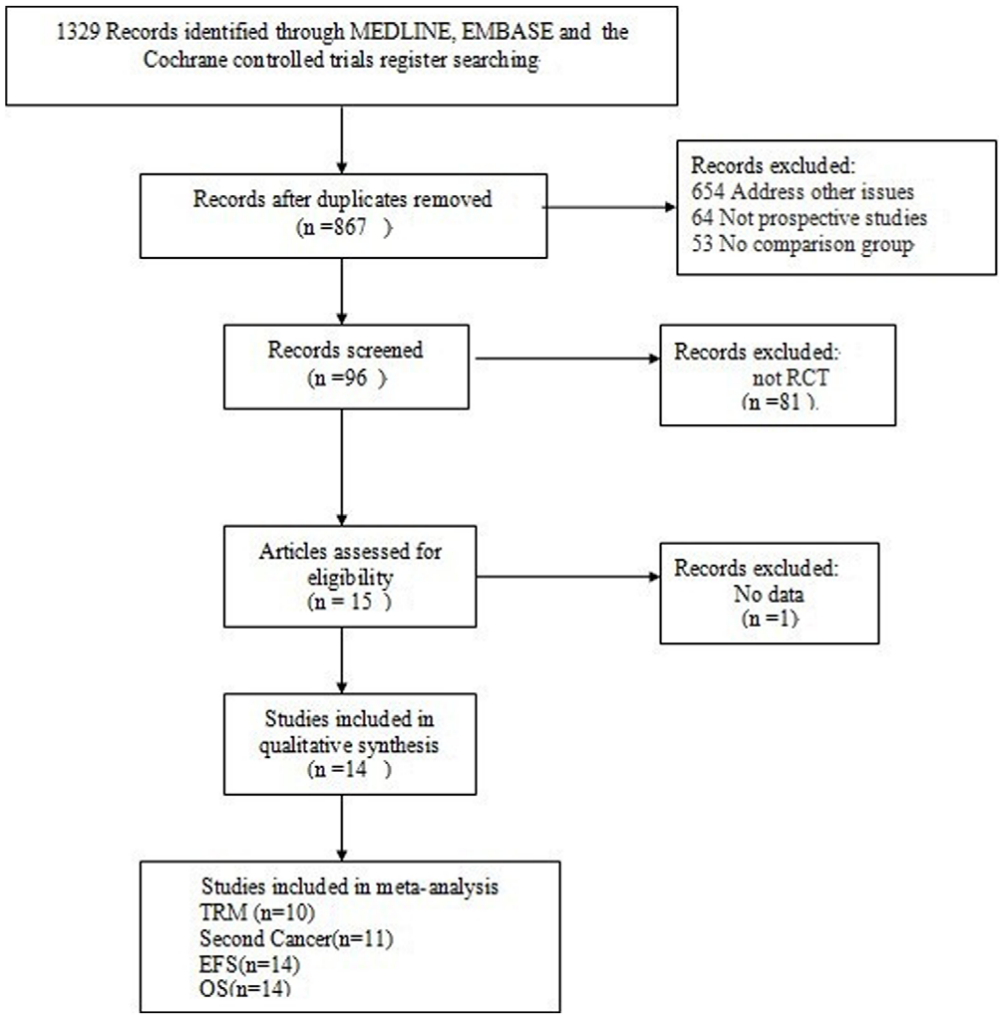
Process of identification and selection of the relevant randomized, controlled trials according to the PRISMA statement.

The process of identification and selection of the relevant randomized controlled trials (RCT) according to the PRISMA statement is depicted in Figure 1. Since the late 1990s, a total of 15 randomized trials in high-risk primary breast cancer of HDCT have been described [10]–[24]. Fourteen of these trials were used in our analysis [10]–[23] with six of them updated after longer follow-up [25]–[30], including 5747 patients (2897 patients treated with HDT/ASCT, and 2850 control patients). Gianni *et al.*'s study [24] was excluded from this analysis because of an insufficient amount of data. All included trials are available as fully published papers. Table 2 shows the characteristics of the trials included. Study quality is shown in Table 3. We also did not explicitly score the methodological quality of the included trials, because the value of doing so is controversial [31].

**Table 2:** Study characteristics.

Study ID	Number of patients	Enrollmentperiod	Number of positive nodes	MedianFollow-Up(years)	Median Age	
					HDC	SDC
IBCSG [10]	344	1995–2000	≥5	8.3	46	46
SBG [15]	525	1994–1998	>5–8	5	48	48
ICCG [11]	281	1993–2001	≥4	5.6	46	48
MDACC [13]	78	1990–1997	≥10 or ≥4 afterchemotherapy	12	45	46
ACCOG [5]	605	1995–1999	≥4	6	45	46
SWOG [16]	536	1996–2001	≥4	5.8	NR	
WSG [17]	403	1995–2002	≥10	4	48	48
CALGB [6]	785	1991–1998	≥10	5.1	44	44
PEGASE 01 [18]	314	1994–1998	≥4	2.75	46	46
NWAST [14]	885	1993–1999	≥4	7	46	45
Dutch pilot [7]	81	1991–1995	Axillary level IIIinvolvement	6.9	45	48
ECOG [8]	511	1991–1998	≥10	6.1	45	43
JCOG [12]	97	1993–1999	≥10	5.25	46	47
GABG [9]	302	1993–2000	≥10	6.1	NR	

ER positive		PR positive		Premenopausal		HDC regimen	SDC regimen	Included in analysis
HDC	SDC	HDC	SDC	HDC	SDC			
46%	41%	NR		67%	67%	EC×3	A/EC×4,CMF×3	TRM, SC, EFS, OS
49.6%(HRS)	54.2%(HRS)	NR		NR		FEC×3/4,CTCb	FEC×9	TRM, SC, EFS, OS
64%	74%	46%	56%	68%	72%	FEC×3,CTCb	FEC×5	TRM, SC, EFS, OS
51%	49%	NR		NR		CAF×8,CEP×2	CAF×8	TRM, SC, EFS, OS
28%	35%	NR		NR		A×4, CT	A×4CMF×8	TRM, SC, EFS, OS
71%(HRS)	61%(HRS)	NR		72%	64%	CA×4, CPCa/T	CAP×3	TRM, SC, EFS, OS
59%	64%	61%	63%	51%	50%	EC×2,CET×2	EC×4,CMF×3	TRM, SC, EFS, OS
69%(HRS)	79%(HRS)	NR		70%	69%	CAF×4,CPCa	CAF×4,CPCa	TRM, SC, EFS, OS
69%	69%	NR		68%	68%	FEC×4CMMp	FEC×4	TRM, SC, EFS, OS
65%	65%	53%	54%	83%	83%	FEC×4,CPT	FEC×5	TRM, SC, EFS, OS
17%	15%	12%	7.5%	76%	84%	FEC×4,CP	FEC×4	TRM, SC, EFS, OS
61%	59%	62%	57%	72%	72%	CAF×6,CT	CAF×6	TRM, SC, EFS, OS
51%	60%	45%	52%	78%	71%	CAF×6,CT	CAF×6	TRM, SC, EFS, OS
61%	60%	58%	68%	55%	62%	EC×4,CMT	EC×4,CMF×4	TRM, SC, EFS, OS

ECOG, Eastern Collaborative Oncology group; GABG, German Autologous Bone Marrow Transplant group; IBCSG, International Breast Cancer Study group; ICCG, International Collaborative Cancer group; JCOG, Japan Clinical Oncology group; MDACC, MD Anderson Cancer Center; NWAST, Netherlands Working Party on Autologous Transplantation in Solid Tumors; SBG, Scandinavian Breast group; SWOG, South Western Oncology group; WSG, West German Study group; EC: epirubicin, cyclophosphamide; AC: doxorubicin, cyclophosphamide; CMF: cyclophosphamide, methotrexate, fluorouracil; FEC: fluorouracil, epirubicin, cyclophosphamide; CTCb: cyclophosphamide, thiotepa, carboplatin; CAF: cyclophosphamide, doxorubicin, fluorouracil; CEP: cyclophosphamide, etoposide, cisplatin; A: doxorubicin; CT: cyclophosphamide, thiotepa; CMF: cyclophosphamide, methotrexate, fluorouracil; CMT: cyclophosphamide, mitoxantrone, thiotepa; CAP: cyclophosphamide, doxorubicin, paclitaxel; CPCa/T: cyclophosphamide, cisplatin, carmustine/thiotepa; CP: cyclophosphamide, cisplatin; CET: cyclophosphamide, epirubicin, thiotepa; CMMp: cyclophosphamide, mitoxantrone, melphalan; HRS: hormone receptor status.

**Table 3:** Study Quality.

Study ID	Secure randomisation	Concealed allocation	Intention to treat	Inclusion and exclusion criteria defined	Extent of follow-up described clearly	Balanced prognosis
IBCSG	Yes	Yes	Yes	Yes	Yes	Yes
SBG	Yes	Yes	Yes	Yes	Notstated	Yes
ICCG	Yes	Yes	Yes	Yes	Notstated	Yes
MDACC	Yes	Yes	Yes	Yes	Notstated	Yes
ACCOG	Yes	Yes	Yes	Exclusioncriteria notstated	Yes	Yes
SWOG	Method notstated	Notstated	Notstated	Yes	Notstated	Yes
WSG	Yes	Yes	Yes	Yes	Notstated	Yes
CALGB	Method notstated	Notstated	Yes	Yes	Notstated	Yes
PEGASE 01	Method notstated	Notstated	Yes	Notstated	Notstated	Yes
NWAST	Yes	Yes	Yes	Yes	Notstated	Yes
Dutch pilot	Yes	Yes	Yes	Exclusioncriteria notstated	Yes	Yes
ECOG	Method notstated	Notstated	Yes	Yes	Notstated	Yes
JCOG	Yes	Yes	Yes	Yes	Yes	Yes
GABG	Yes	Yes	Yes	Yes	Notstated	Control arm had less women with <16+ve nodes, small tumours

### Meta-analysis

**Figure 2:**
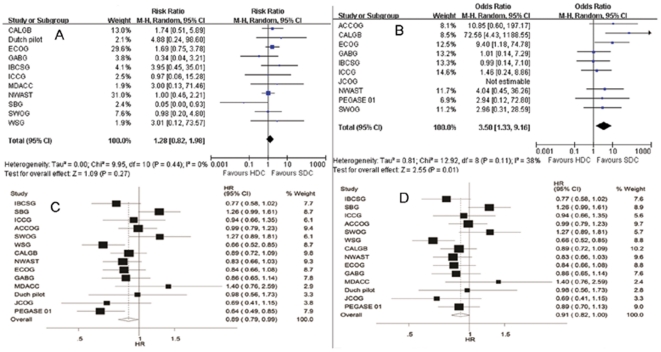
Forest plot of the RR/HR. The size of the squares reflects each study's relative weight and the diamond (◊) represents the aggregate RR/HR and 95% CI. (A) Second cancers; (B) Treatment-related mortality; (C) Disease-free survival; (D) Overall survival.

The overall results of this analysis are shown in Figure 2. TRM was reported in 10 studies [10], [11], [13]–[17], [19], [21], [23]. Patients randomly assigned to HDCT had a statistically significantly greater risk of death than patients randomly assigned to receive chemotherapy only. More deaths were found among the patients assigned to HDCT than among the patients who received chemotherapy only (RR = 3.42; 95% CI = 1.32–8.86). Eleven studies reported the risk of second (non-breast) cancers [11]–[16], [18]–[22]. The risk of second (non-breast) cancers was not significantly different in the group assigned to HDCT from the group assigned to chemotherapy only (RR = 1.28; 95% CI = 0.82–1.98). DFS data were available for 14 studies [10]–[23]. DFS was better with HDCT than with chemotherapy only (HR = 0.89; 95% CI = 0.79–0.99). Of the 14 studies for which overall survival data were available [10]–[23], the difference in overall survival was not statistically significant [(HR = 0.91; 95% CI = 0.82–1.00), *p* = 0.062].

### Subgroup analysis

Protocol as described in the methods section, the studies were summarized in subgroups according to a cut off value regarding certain characteristics. The subgroup analysis was performed according to a variety of criteria, and the outcome is shown in Table 4.

**Table 4:** Subgroup analysis according to patient characteristics.

patient characteristics		Second Cancer (RR)			TRM(RR)			EFS			OS		
		RR(95%CI)	H		RR(95%CI)	H		HR(95%CI)	H		HR(95%CI)	H	
			Q	*P*		Q	*P*		Q	*P*		Q	*P*
All		1.28(0.82–1.98)	9.95	0.44	3.50(1.33–9.16)	12.92	0.11	0.89(0.79–0.99)	28.59	0.007	0.91(0.82–1.00)	23.07	0.041
Median age <47 in each group	+	1.47(0.90–2.40)	2.08	0.72	5.91(1.60–21.89)	8.56	0.13	0.85(0.75–0.96)	9.16	0.165	0.88(0.80–0.97)	4.57	0.601
	−	0.88(0.11–7.10)	6.00	0.11	1.46(0.24–8.86)	NA	NA	0.89(0.66–1.20)	14.64	0.006	0.89(0.66–1.20)	14.56	0.006
PR positive rate >50% in each group	+	1.26(0.79–2.00)	2.82	0.83	3.89(1.26–12.05)	10.52	0.1	0.82(0.73–0.93)	13.69	0.090	0.85(0.77,0.93)	10.78	0.292
	−	1.36(0.15–12.43)	7.65	0.05	2.62(0.23–29.21)	1.96	0.16	1.03(0.83–1.26)	7.83	0.098	1.11(0.95–1.29)	2.81	0.422
Premenopausal rate >70% in each group	+	1.36(0.84–2.21)	1.86	0.76	11.88(2.13–66.16)	3.27	0.20	0.89(0.79–1.01)	5.49	0.359	0.89(0.79–1.01)	5.49	0.359
	−	1.32(0.38–4.64)	2.69	0.44	1.27(0.45–3.61)	0.4	0.94	0.75(0.65–0.86)	4.78	0.311	0.80(0.71–1.37)	4.21	0.378

NA: not applicable.

### Publication bias

**Figure 3:**
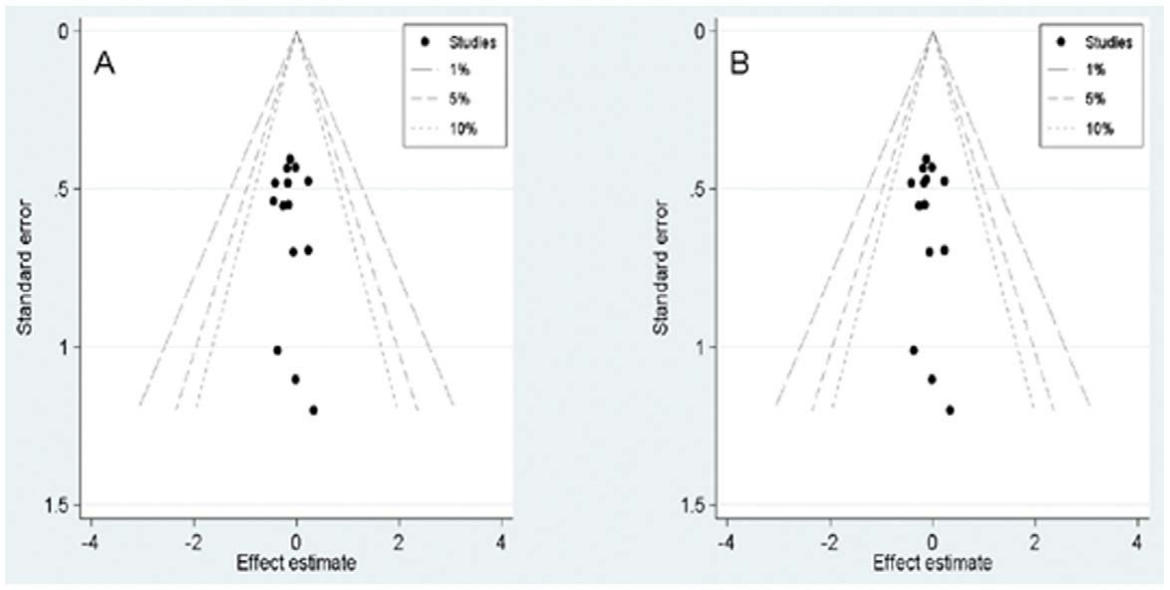
Contour-enhanced funnel plot for publication bias test. (A) Disease-free survival; (B) Overall survival.

Begg's funnel plot and Egger's test were performed to assess the publication bias in the literature. All studies investigating DFS yielded a Begg's test score of *p* = 0.547 and an Egger's test score of *p* = 0.609. According to the contour-enhanced funnel plot (Figure 3), publication bias was not found in any study. Similar results were found for OS (*p* = 0.622 and 0.540). The contour-enhanced funnel plot suggests no presence of publication bias for DFS and OS.

## Discussion

This meta-analysis combines the results from fourteen methodologically satisfactory trials that prospectively enrolled and randomly assigned 5747 patients with high-risk primary breast cancer to therapy with either HDCT or conventional chemotherapy. Our pooled results suggests that HDCT is associated with a modest advantage in DFS ((HR = 0.89; 95% CI = 0.79–0.99) but not in OS (HR = 0.91; 95% CI = 0.82–1.00, *p* = 0.062). However, it was found that age and hormone receptor status had a significant interaction with prolonged OS.

Retrospective research had suggested that HDCT in high-risk primary breast cancer demonstrated significant favorable outcomes compared with historical data, but a prospective randomized clinical trial is the accepted standard for comparing different treatments such as HDCT for high-risk primary breast cancer. In fact, the results of prospectively randomized clinical trials were conflicting. Finally, other trials have shown nonsignificant trends in favor of HDCT. Only in the WSG trial, which employed tandem HDCT, did the EFS advantage translate into an OS benefit [22]. An important factor that might account for the superiority of HDCT in the WSG trial is the double autologous stem-cell transplantation. High-dose therapy with tandem autologous stem-cell rescue is effective for treating high-risk neuroblastoma [32] and multiple myeloma [33] with encouraging long-term survival. If we excluded the WSG trial [22] from our meta-analysis, the final conclusion that age and hormone receptor status had a significant interaction with prolonged OS would not have changed (data not shown).

In an effort to shed some light on the impact of HDCT as a first-line treatment for high-risk primary breast cancer, the data were pooled from available published trials for meta-analysis. However, two previous meta-analyses [5], [6] provided different evidence of the impact of HDCT on high-risk primary breast cancer outcome. The combined HRs, which are the preferred summary statistics for reporting time-to-event data [8], were not used in the previously published meta-analyses [5]. The most widely recommended approach for summarizing the effect of treatment from time-to-event data in clinical trials is to use a hazard ratio. The best statistic to use is the hazard ratio (HR) in Meta-analyses of published time-to-event outcomes. HRs given in trial reports can be used directly, or if sufficient summary statistical information or Kaplan-Meier curves are presented, then HRs can be estimated indirectly [8].

Another meta-analysis was based on individual patient data (IPD) for the OS. For time-to-event outcomes, the gold standard approach is to obtain IPD from each included study. IPD should overcome problems of within-study selective reporting [34] and should allow a more complete analysis including the potential to investigate treatment–covariate interactions [35]. IPD meta-analyses are difficult to perform because of challenges in collecting the patient-level data. This analysis was displayed as an abstract, and no details were shown. We could get the data of median age, PR status and menopausal (MP) status of the whole population [6], so we used a median age of 47, a PR positive rate of 50% and a premenopausal rate of 70% as cutoff values to complete the subgroup analyses. Therapeutic strategies are generally based on the endocrine responsiveness and the estimated risk of relapse defined by tumor size, axillary lymph node involvement, histologic and nuclear grade, lymphatic and/or vascular invasion, HER2/neu-overexpression and age [36]. Our pooled results suggest that HDCT is associated with a modest advantage in DFS; however, the EFS advantage did not translate into an OS benefit. When we subgrouped the studies according to age and hormone receptor status, we found a prolonged OS while performing HDCT in high-risk primary breast cancer. The analyses of the Dutch [12] and WSG [22] trials now suggest a predictive value for HDC benefit for HER2-negative and triple-negative (ER/PR/HER2-negative) status [37]. The hypothesis-generating observations suggest that this breast cancer category presents increased sensitivity to dose intensification of alkylating agents and should remain the subject of clinical HDCT studies [37].

Treatment-related-mortality (TRM) may account for our finding that improved DFS did not translate into improved OS. Patients who received HDCT had a greater risk of dying during remission than patients who received non-myeloablative chemotherapy, primarily because of the toxicity associated with the regimen resulting in patients' protracted pancytopenia, which results in a prolonged risk of infection or bleeding. Although the DFS may be prolonged by HDCT, the benefit was offset in part by treatment-related deaths. Two studies reported highly significant TRM during HDCT [11], [13], which was higher than HDCT in other studies. A considerable reduction in TRM would be needed to demonstrate a survival benefit from HDCT. Women 50 years and older appeared to have a higher risk of TRM than younger women if randomly assigned to HDCT [11]. Young age will result in a reduced TRM.

Heterogeneity is a potential problem when interpreting the results of a meta-analysis. However, no evidence for statistically significant heterogeneity was found in any of the models used. This result indicates that using an overall estimation of the value of HDCT may be appropriate. To eliminate heterogeneity, we divided the 14 studies into subgroups as far as possible; subsequently, heterogeneity decreased for subgroups of age and hormone receptor status, which revealed that most of the studies could not be grouped helpfully according to age and hormone receptor status.

Quality assessment was based on the reporting of the study methods and results, namely: randomisation, allocation concealment, intention to treat, defined inclusion and exclusion criteria, extent of follow-up described clearly, balanced prognosis. Ad hoc scores may lack demonstrated validity, and the results may not be associated with quality [38]. Overall, these studies included in the analysis were considered of good quality, typically prospective multicenter trials that reported outcomes analyzed as ITT; were performed at the national level; and were published in peer-reviewed journals.

From our analysis, age and hormone receptor status seem to have a significant interaction with prolonged OS. However, we must explicitly state that caution is highly advisable when interpreting subgroup analyses. These cannot be used for recommendations on treatment selection for individual patients. Nevertheless, with appropriate care, they can be used in the development of new, empirically based research.

Several limitations should be considered when interpreting the results of our analysis. First, our results were based on unadjusted estimates, while a more precise analysis could be conducted if individual data were available, which would allow for adjustment by other co-variates. Second, this analysis does not use primary patient data, but rather relies on information available in prior publications. Because of the lack of original data, one RCT was excluded because no information on HR was available [24]. Third, only published studies were included in this meta-analysis. Nonsignificant or negative findings may be unpublished. Farquhar *et al.* [5] reported 6 ongoing RCT of HDCT in primary breast cancer; however, we only found two such articles. In addition, our analyses did not clarify whether double unit grafts would influence the outcome of HDCT in primary breast cancer.

In conclusion, HDCT has a benefit on DFS and OS compared to SDC in some special patients with high-risk primary breast cancer. Because of the limitations that we mentioned above, our results may not be used as a guideline for primary breast cancer treatment. Further study is needed to determine whether specific subgroups of patients, such as those who are HER2-negative or triple-negative, also benefit from HDCT. Alternatively, these questions could be addressed by combining individual patient data from the completed trials, but such an endeavor would require a large investment of resources as well as multinational cooperation.

## Supporting Information

Checklist S1 **PRISMA Checklist.**
(PDF)

Protocol S1 **High-dose chemotherapy followed by autologous stem cell transplantation as a first-line therapy for high-risk primary breast cancer (Protocol).**
(DOC)
