# Correction: High-Dose Chemotherapy Followed by Autologous Stem Cell Transplantation as a First-Line Therapy for High-Risk Primary Breast Cancer: A Meta-Analysis

**DOI:** 10.1371/annotation/928a7cff-1ae7-4ce5-908c-f765ea53663f

**Published:** 2012-11-16

**Authors:** Jing Wang, Qiguo Zhang, Rongfu Zhou, Bing Chen, Jian Ouyang

Our article reports a meta-analysis of available evidence on high-dose chemotherapy followed by autologous stem cell transplantation for primary breast cancer. A previous publication by Berry et al. reported a closely related meta-analysis and the authors regret not citing this article:

Berry DA, Ueno NT, Johnson MM, et al. High-dose chemotherapy with autologous stem cell support as adjuvant therapy in breast cancer: Overview of 15 randomized trials. J Clin Oncol 2011;29:3214-3223

Berry et al. provide evidence that high-dose chemotherapy followed by autologous stem cell transplantation (HDCT) support prolongs relapse-free survival but does not improve overall survival in patients with high-risk primary breast cancer. In our subgroup analysis, age and hormone receptor status had a significant interaction with OS. In the study by Berry et al., OS was statistically different by treatment arm in the subgroup for women with both hormone receptor-negative and HER2-negative tumor, for whom there was a 33% reduction in the risk of death. The hypothesis suggest that this breast cancer category presents increased sensitivity to dose intensification of alkylating agents and should remain the subject of clinical HDCT studies.

While our meta-analysis evaluated publication bias and heterogeneity in the included studies, our analysis did not include an analysis of individual patient data, which Berry et al. did carry out. The lack of an analysis on individual patient data can be considered as a limitation of our meta-analysis. 

