# Supplementary material for: High-Dose Chemotherapy Followed by Autologous Stem Cell Transplantation as a First-Line Therapy for High-Risk Primary Breast Cancer: A Meta-Analysis
Source: PLoS One. 2012 Mar 12;7(3):e33388. doi: 10.1371/journal.pone.0033388 (PMC3299795; doi:10.1371/journal.pone.0033388)
Supplement: Protocol S1 — High-dose chemotherapy followed by autologous stem cell transplantation as a first-line therapy for high-risk primary breast cancer (Protocol). (DOC) [file pone.0033388.s002.doc]

**High-dose chemotherapy followed by autologous stem cell transplantation as a first-line therapy for high-risk primary breast cancer (Protocol)**

**A B S T R A C T**

This is the protocol for a review and there is no abstract. The objectives are as follows:

The main objective of this review is to find the best available treatment with high-dose chemotherapy followed by autologous stem cell transplantation (ASCT) for patients with primary breast cancer. We will analyse overall survival (OS) and disease-free survival (DFS) as the primary endpoint; and will also analyse progression-free survival (PFS), response rate, treatment-related mortality (TRM), and second (non-breast) cancers as secondary endpoints.

**B A C K G R O U N D**

**Description of the condition**

In 2010, breast cancer was ranked first in cancer incidence among women in US, with an estimated 207,090 cases. For cancer-related mortal­ity, breast cancer was ranked second among women, with an estimated 39,840 deaths. Patients with stage III breast cancer or patients with stage II breast cancer and multiple positive axillary lymph nodes have an approximately 80% relapse rate at 5 years if treated only with locoregional therapy*.*

**Description of the intervention**

One of the strategies to improve the outcome for high- risk patients was to increase the dose of chemotherapy to enhance its cytotoxicity. The technique of high-dose chemotherapy followed by autologous stem cell transplantation (HDCT) has been considered an exciting development because, by addressing the problem of bone-marrow toxicity, it permits the administration of doses many times higher than could otherwise be considered and thus results in the death of more tumor cells. Therefore, most research on breast cancer management focuses on improving breast cancer outcomes in this area.

**Why it is important to do this review**

Many randomized trials performed by several institutions across the world have addressed conflicting results regarding the benefit of HDCT for primary breast cancer. A large meta-analysis of 13 randomized trials including 5,064 women, showed a significant benefit in event-free survival for the HDCT group, and overall survival rates were not significantly different at any stage of follow-up. However, individual patient data from 15 known randomized trials including 6,210 patients showed a modest improvement in OS (HR 0.89; 95% CI 0.81–0.98; P=0.016) for the HDCT group compared with standard dose chemotherapy (SDC). It became unclear whether HDCT results in a survival benefit compared with SDC. To arrive at comprehensive estimates of the survival benefit from the totality of the data available.

**O B J E C T I V E S**

The main objective of this review is to find the best available treatment with high-dose chemotherapy followed by autologous stem cell transplantation (ASCT) for patients with primary breast cancer. We will analyse overall survival (OS) and disease-free survival (DFS) as the primary endpoint; and will also analyse treatment-related mortality (TRM), and second (non-breast) cancers as secondary endpoints.

**M E T H O D S**

Criteria for considering studies for this review

**Types of studies**

We will accept only RCTs for this review and we will include both, full text and abstract publications.

**Types of participants**

We will include trials on patients with primary primary breast cancer, and we will not apply any restriction on age, gender or ethnicity.

**Types of interventions**

The intervention is defined as follows:

●Any high-dose chemotherapy followed by ASCT for primary patients.

The control interventions are defined as follows.

●Different HDT regimens followed by ASCT.

●Conventional chemotherapy.

●Different dosages or time schedules of the HDT followed by ASCT therapy regimen.

Primary analysis will include studies regarding ASCT versus no ASCT, as far as they are sufficiently similar. If the systematic search identifies clinical studies for other comparisons (e.g. different HDT treatment options), we will meta-analyse them as well.

**Types of outcome measures**

**Primary outcomes**

●Disease-free survival: DFS will be defined as the time interval from random treatment assignment/entry to the study to first progression or relapse, death from any cause or the last follow-up.

●Overall survival: OS will be defined as the time interval from random treatment assignment/entry to the study to death from any cause or to last follow-up.

**Secondary outcomes**

●Treatment-related mortality: TRM will be defined as death related to the intervention under investigation.

●Second (non-breast) cancers: Second cancers will be defined as non-breast cancer related to the intervention under investigation.

**Electronic searches**

We will adapt the search strategies suggested in the CochraneHand-book of Systematic Reviews of Interventions To reduce language bias, we will not apply language restriction.

We will search the following databases/sources of medical literature:

● Cochrane Controlled Trials Register.

● MEDLINE.

● EMBASE.

● American Society of Hematology (ASH).

● American Society of Clinical Oncology (ASCO).

● European Haematology Association (EHA).

**Data collection and analysis**

**Selection of studies**

After the first reviewof all titles and abstracts of the identified studies from the above sources, two review authors will independently reject all studies that are clearly ineligible. We will assess selected studies by using an eligibility form regarding study design and compliance with inclusion criteria. If there is any doubt, we will include full text analysis and discuss eligibility with both review authors to finalise a decision. We prefer to include studies rather than to lose relevant data. According to PRISMA,

we will use a flow chart to document the study selection process, showing the total numbers of retrieved references, the numbers of included and excluded studies.

**Data extraction and management**

Two review authors will independently extract data according to Chapter Seven of the Cochrane Handbook for Systematic Reviews of Interventions by using the standardised data extraction form with following items.

●General information: author, title, source, publication date, country, duplicate publications.

●Quality assessment: allocation concealment, blinding (participants, personnel, outcome assessors), incomplete outcome data, selective outcome reporting, other sources of bias.

●Study characteristics: trial design, aims, setting and dates, source of participants, inclusion/exclusion criteria, comparability of groups, subgroup analysis, statistical methods, power calculations, treatment cross-overs, compliance with assigned treatment, length of follow-up, time point of randomisation.

●Participant characteristics: age, gender, ethnicity, number of participants recruited/allocated/evaluated, participants lost to follow-up.

●Interventions: setting, type of (multi-agent) chemotherapy (intensity of regimen, number of cycles), transplantation with or without growth factor support, transplant details, infection prophylaxis, duration of follow-up.

●Outcomes: overall survival, progression-free survival, treatment-related mortality, second cancers

**Assessment of Quality of included studies**

To assess the methodological quality, we will use a questionnaire

for the following criteria.

● Randomisation.

● Allocation concealment.

● Intention to treat.

● Inclusion and exclusion criteria defined.

● Extent of follow-up described clearly.

● Balanced prognosis.

Our judgement of the review will involve an answer for each criteria.

**Measures of treatment effect**

For binary outcomes, we will calculate risk ratios (RR) with 95%

confidence intervals (CI) for each trial and for continuous outcomes. For time-to-event outcomes, we will extract hazard ratio (HR) from published data.

**Assessment of heterogeneity**

We will pool all results by applying meta-analysis using a fixed-effect model.We will assess heterogeneity of treatment effects between trials by using a CHI^2^- test with a significance level at P <0.1. We will use the I² statistic to quantify possible heterogeneity (I² > 30% moderate heterogeneity, I² > 75% considerable heterogeneity). We will explore the potential causes of heterogeneity by subgroup analysis.

**Assessment of reporting biases**

In meta-analyses with at least ten trials, we will explore potential publication by generating a funnel plot and statistically tested by using the methods of Egger et al. and Begg et al. We will consider P < 0.1 significant for the aim of this test.

**Data synthesis**

We will use the Cochrane statistical package Review Manager (RevMan) 5.1 and STATA 10.0 for analysis. One review author will input data into software and a second review author will check it for accuracy.

**Subgroup analysis and investigation of heterogeneity**

The median patient age was 46 years, hormone receptor (PR) status was positive in 46.8% of patients, and 68.9% of patients were premenopausal in the overall population. If appropriate, we will consider performing subgroup analyses on the following characteristics.

(a) Median age <47 in each group;

(b) PR positive (positive if either estrogen or progesterone receptor was positive) rate >50% in each group;

(c) Premenopausal rate >70% in each group.
